# Association between the Clinical Frailty Scale and Neurological Outcomes in Out-of-Hospital Cardiac Arrest: A Retrospective Study

**DOI:** 10.31083/RCM26333

**Published:** 2025-03-21

**Authors:** Haw Hwai, Chien-Kai Wu, Chien-Yu Chi, Min-Shan Tsai, Chien-Hua Huang

**Affiliations:** ^1^Department of Emergency Medicine, National Taiwan University Hospital, National Taiwan University Medical College, 100 Taipei; ^2^Department of Emergency Medicine, Taipei City Hospital, 111 Taipei; ^3^Department of Emergency Medicine, National Taiwan University Hospital Yun-Lin Branch, National Taiwan University Medical College, 640 Douliu

**Keywords:** clinical frailty scale, out-of-hospital cardiac arrest, neurological outcomes

## Abstract

**Background:**

Frailty is a physical condition characterized by increased vulnerability to external stressors. This study investigated the impact of premorbid frailty, as measured by the Clinical Frailty Scale (CFS), on neurological prognosis in patients with out-of-hospital cardiac arrest (OHCA).

**Methods:**

This is a single-center retrospective study. Data from 2006 to 2020 were analyzed for 595 adult OHCA patients admitted to the intensive care unit of National Taiwan University Hospital following resuscitation. Variables included demographics, medical history, resuscitation details, post-resuscitation data, and frailty assessments based on CFS. The primary outcome was favorable neurological performance, defined as a cerebral performance category (CPC) score of 2 or less at discharge.

**Results:**

In total, 523 of the 595 patients were included in the analysis. Among these, 224 survived, and 173 exhibited favorable neurological outcomes. Patients with favorable outcomes had significantly lower CFS scores than those with poor outcomes (3.2 ± 1.5 vs. 4.5 ± 1.8, *p* < 0.0001). The proportion of favorable neurological outcomes declined as CFS scores increased. Multivariate logistic regression analysis identified several factors independently associated with worse neurological outcomes: CFS >4 (odds ratio (OR): 0.301, 95% confidence interval (CI): 0.163–0.540), age >70 years (OR: 0.969, 95% CI: 0.953–0.986), history of malignancy (OR: 0.421, 95% CI: 0.209–0.813), epinephrine >2 mg during resuscitation (OR: 0.776, 95% CI: 0.712–0.840), and arterial blood gas pH <7.1 (OR: 28.396, 95% CI: 6.487–129.350). The model demonstrated good performance, with an area under the curve (AUC) value of 0.853. No significant relationships were observed between CFS and other variables.

**Conclusions:**

CFS values ≤4 were independently associated with favorable neurological outcomes following OHCA.

## 1. Introduction

### 1.1 Out-of-Hospital Cardiac Arrest

Out-of-hospital cardiac arrest (OHCA) is the leading cause of mortality worldwide, with an incidence rate of 30–60 per 100,000 person-years [1]. Despite efforts to improve outcomes, e.g., promoting bystander cardiopulmonary resuscitation (CPR), strengthening emergency medical services, and advancing post-resuscitation care, the survival-to-discharge rate of OHCA remains poor [2]. A proportion of cardiac arrest survivors experience neurological sequelae, including cognitive dysfunction or persistent vegetative state [3].

Resuscitation and post-resuscitation care are both costly and labor-intensive [4]. Therefore, accurately predicting the prognosis of OHCA can help guide critical decisions regarding the continuation of resuscitation and withdrawal of life-sustaining treatment [5]. Key outcome indicators in medical intensive care unit (ICU) patients include age, the severity of acute illness, and underlying comorbidities [6, 7, 8]. Notably, while older patients typically exhibit low rates of successful resuscitation and favorable neurological outcomes, a substantial number survive with good cerebral performance after discharge [8].

### 1.2 Current Model for Predicting Neurological Outcomes 

Several methods have been utilized to predict neurological outcomes, including clinical assessments, biomarkers, electrophysiological studies, and brain imaging. However, none of these methods are sufficient when used in isolation [9]. Existing models, such as the OHCA score, CaRdiac Arrest Survival Score (CRASS), and the Cardiac Arrest Hospital Prognosis (CAHP) score, integrate demographic information, CPR variables, and laboratory data, demonstrating strong predictive performance [10, 11, 12]. Nevertheless, these models are limited in applicability before the return of spontaneous circulation (ROSC). Additionally, their reliance on laboratory data and resuscitation variables complicates effective communication with the patient’s family during decision-making processes. The Clinical Frailty Scale (CFS), which relies solely on history-taking, offers a practical and comprehensive overview of the patient’s condition, aligning closely with clinical intuition. Moreover, the simplicity and alignment of the CFS make it a promising tool for objectifying “clinical judgment” in such scenarios.

### 1.3 Frailty

In geriatric medicine, frailty describes an increased vulnerability to external stressors [9]. Beyond age, frailty independently predicts mortality [9]. Frailty is measured through various methods, including the frailty phenotype and frailty index [9]. The frailty phenotype encompasses physical conditions such as weight loss, self-reported exhaustion, slow gait speed, decreased muscle strength, and low physical activity; three or more conditions indicate frailty [9]. In contrast, the frailty index integrates deficits across multiple domains to quantify frailty levels, such as symptoms, abnormal laboratory values, diseases, and disabilities [9].

The CFS is a clinical judgment tool closely associated with the frailty index [9]. Widely used in critical care research, the CFS reliably predicts patient outcomes due to its convenience [9]. Further, the CFS considers medical and functional conditions, including activity levels, underlying medical conditions, dependence on daily activities, and dementia. Initially, the CFS employed a seven-point scale, ranging from one (very fit) to seven (severely frail), as introduced by the Canadian Study on Health and Aging [9]. In 2007, the scale was expanded to include two end-of-life points: CFS 8 (severe frailty) and CFS 9 (terminally ill). However, recent study indicated no significant differences in neurological outcomes among patients with CFS scores of 5 or higher [13]. Consequently, it is appropriate to group patients with CFS scores exceeding seven, consistent with the original seven-point scale.

### 1.4 Study Aim

This study aimed to determine the association between the degree of frailty and the neurological prognoses of OHCA survivors. To enhance understanding of the role of frailty in OHCA, this study incorporated a CFS assessment with other significant factors into a risk stratification model using multiple logistic regression.

## 2. Material and Methods

### 2.1 Study Design and Population

This study involved a secondary database analysis containing records of prospectively recruited adult OHCA patients.

The inclusion criteria were as follows: adult OHCA patients aged over 20, patients who achieved a ROSC, patients who survived and were admitted to the ICU, and patients admitted between January 1, 2006, and December 31, 2020. Conversely, the exclusion criteria included traumatic OHCA patients and patients or family members who signed do-not-resuscitate (DNR) forms before or during CPR.

### 2.2 Study Setting

The study was conducted at the National Taiwan University Hospital, a leading tertiary medical center and teaching hospital in Taipei, Taiwan, equipped with 150 ICU beds. The emergency department handles approximately 100,000 patients annually. To optimize resuscitation, healthcare providers perform CPR using the advanced cardiac life support (ACLS) teamwork model, typically involving 4 to 7 personnel assigned specific roles such as airway management, chest compressions, defibrillation, medication administration, and leadership. For OHCA patients who do not achieve ROSC, CPR is performed for at least 30 minutes unless a do-not-resuscitate order has been documented. Based on ultrasonography findings and blood gas analysis, physicians treat reversible causes such as hemorrhage, cardiac tamponade, and metabolic acidosis. Extracorporeal membrane oxygenation CPR is employed for OHCA patients with refractory shockable rhythms.

### 2.3 Data Collection

Collected data included age, gender, and pre-existing systemic diseases categorized using Charlson’s comorbidity index [14]. Core resuscitation elements were registered using the Utstein style [15]. Information such as CPR duration, epinephrine dosage during CPR, post-ROSC vital signs, and laboratory values (including arterial blood gas, lactic acid, and white blood cell counts) was recorded upon ICU admission and analyzed.

### 2.4 Frailty Measurement

Frailty was assessed by physicians using the seven-point CFS through a review of medical and nursing records. Guided utilizing the framework for retrospective chart reviews by Worster *et al*. [16], this evaluation involved 20 emergency medicine physicians anonymized to the study hypothesis and the patient’s outcomes at discharge. Before the study, physicians were trained on the CFS and the cerebral performance category based on its 2005 original version.

Due to the reliance of the CFS on clinical judgment of baseline cognitive function, mobility, and underlying medical conditions, admission notes incorporated baseline condition information. These data were gathered from caregivers or family members during history-taking and from patients’ comprehensive electronic medical records, including details from prior hospital admissions, outpatient visits, and diagnostic reports. Patients with incomplete histories or insufficient data to assess the CFS accurately were excluded from the study. This study did not conduct an inter-rater reliability assessment of the CFS, citing prior research indicating substantial reliability of the CFS, and the primary outcome was good cerebral performance at hospital discharge, defined as a cerebral performance category (CPC) in emergency department settings [15]. Mean values were used to interpolate missing laboratory or vital sign data, as missing data occurred in less than 5% of cases. Ethical considerations and data abstraction methods were approved by the Institutional Review Board (IRB) of the affiliated university. Data were anonymized, coded using identification numbers, and stored securely.

### 2.5 Outcome Measurement

The primary outcome was good cerebral performance at hospital discharge, defined as a CPC score of 1 or 2. Neurological outcomes were categorized into five CPC levels ranging from 1 (conscious with normal function or slight disability, capable of work) to 5 (brain death state). Intermediate levels included CPC 2 (conscious with moderate disability, able to perform daily activities independently), CPC 3 (conscious with severe disability, dependent on others for daily activities), and CPC 4 (comatose or in a vegetative state). For this study, favorable neurological outcomes were defined as CPC scores of 1 or 2 [17].

### 2.6 Statistical Analyses

Univariate analyses of patient characteristics, pre-existing conditions, resuscitation events, post-ROSC vital signs, and laboratory data were conducted using a student’s *t*-test and chi-square test. A generalized additive model (GAM) identified the CFS threshold. Factors with a value of *p* < 0.1 in univariate analysis were included in multivariate logistic regression (MLR). Representative factors were chosen to address collinearity, such as selecting diastolic blood pressure (DBP) over systolic blood pressure (SBP) and epinephrine dosage over CPR duration. MLR analysis identified independent associations with favorable neurological outcomes and developed a prediction model. The model’s precision was evaluated using receiver operating characteristic curves (ROCs) and the area under the curve (AUC). Statistical analyses were conducted using R version 3.3.1 (R Foundation for Statistical Computing, Vienna, Austria), with *p* < 0.05 indicating statistical significance.

## 3. Results

### 3.1 Patient Selection

**Fig. 1.**
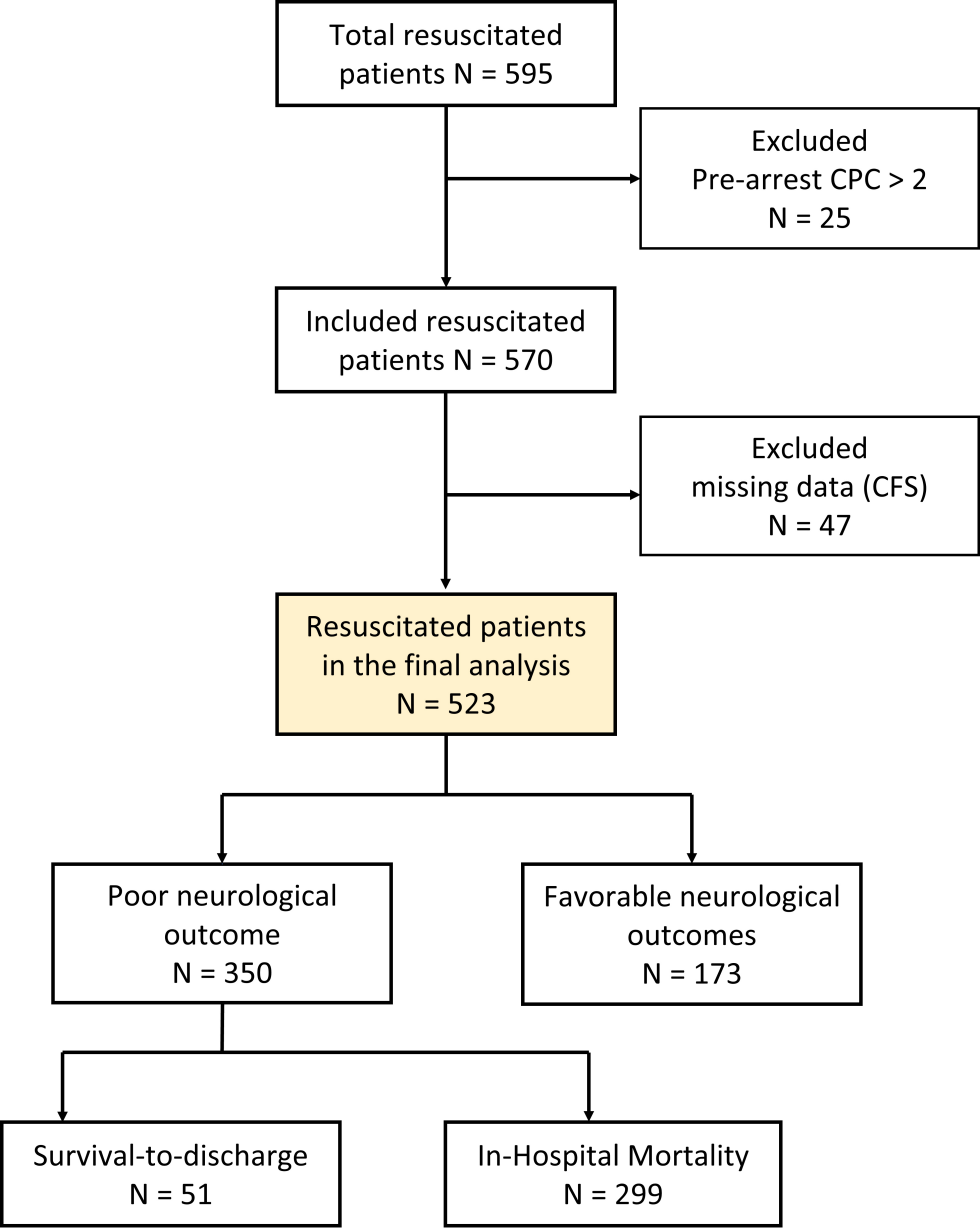
**Flowchart of patient inclusion and exclusion**. This study initially included 595 resuscitated patients. After excluding those with pre-arrest cerebral performance category (CPC) values >2 and missing data, 523 patients remained for analysis. Among these, 350 patients experienced poor neurological outcomes, comprising 51 survivors and 299 non-survivors. In contrast, 173 patients achieved favorable neurological outcomes. CFS, Clinical Frailty Scale.

A total of 595 patients were analyzed. After excluding patients with pre-arrest CPC values greater than two or missing CFS data, 523 patients (224 survivors and 299 non-survivors) were included in the analysis. Among these, favorable neurological outcomes were demonstrated in 173 survivors (Fig. 1).

### 3.2 Demographic and Clinical Characteristics of the Study Population

Patients with favorable neurological outcomes were younger, predominantly male, had lower CFS values, and were more likely to have experienced a witnessed collapse. Compared with poor neurological outcomes, these patients had a lower rate of malignancy among pre-existing systemic diseases, received lower doses of epinephrine, and underwent shorter durations of CPR. Additionally, they exhibited significantly higher SBP and DBP values. Notably, the factors influencing survival and neurological prognosis were highly similar across groups, except for a considerably lower prevalence of chronic obstructive pulmonary disease (COPD) or asthma in the favorable neurological outcomes group, which was not observed in the survival group (Table 1). However, this difference was not statistically significant.

**Table 1.** **Patient characteristics**.

	Survivor	Non-survivor	*p*-value	Favorable neurological outcomes (n = 173)	Poor neurological outcomes (n = 350)	*p*-value
	(n = 224)	(n = 299)				
Age (SD)	60.81 (15.82)	68.56 (15.34)	<0.001	59.17 (15.40)	68.24 (15.45)	<0.001
Gender (male) (%)	177 (79.0%)	197 (65.9%)	0.003	139 (80.3%)	235 (67.1%)	0.002
Clinical Frailty Scale (SD)	3.4 (1.6)	4.6 (1.8)	<0.001	3.2 (1.5)	4.5 (1.8)	<0.001
Witnessed collapse (%)	198 (88.4%)	223 (74.6%)	<0.001	153 (88.4%)	268 (76.6%)	0.002
Prehospital cardiopulmonary resuscitation (%)	191 (85.3%)	256 (85.6%)	1.000	148 (85.5%)	299 (85.4%)	1.000
Malignancy (%)	25 (11.2%)	72 (24.1%)	<0.001	17 (9.8%)	80 (22.9%)	0.001
End-stage renal disease (%)	16 (7.1%)	28 (9.4%)	0.455	11 (6.4%)	33 (9.4%)	0.307
Renal disease (%)	22 (9.8%)	30 (10.0%)	1.000	19 (11.0%)	33 (9.4%)	0.687
Diabetes mellitus (%)	58 (25.9%)	96 (32.1%)	0.148	44 (25.4%)	110 (31.4%)	0.189
Chronic obstructive pulmonary Disease/asthma (%)	13 (5.8%)	28 (9.4%)	0.182	8 (4.6%)	33 (9.4%)	0.080
Heart failure (%)	16 (7.1%)	33 (11.0%)	0.174	12 (6.9%)	37 (10.6%)	0.237
Cerebral vascular accident (%)	21 (9.4%)	38 (12.7%)	0.292	15 (8.7%)	44 (12.6%)	0.238
Hypertension (%)	122 (54.5%)	1978 (59.5%)	0.285	91 (52.6%)	209 (59.7%)	0.146
Coronary artery disease (%)	77 (34.4%)	104 (34.8%)	0.997	59 (34.1%)	122 (34.9%)	0.942
Cardiopulmonary resuscitation duration (min) (SD)	27.29 (19.69)	36.11 (20.07)	<0.001	24.61 (19.27)	36.15 (19.82)	<0.001
Epinephrine (mg) (SD)	2.959 (3.278)	4.634 (4.051)	<0.001	2.451 (3.013)	4.584 (3.987)	<0.001
Heart rate (bpm) (SD)	101.1 (29.4)	104.6 (32.8)	0.210	101.5 (29.6)	103.9 (32.3)	0.400
Systolic blood pressure (mmHg) (SD)	130.6 (45.3)	114.7 (43.0)	<0.001	131.9 (45.3)	116.4 (43.5)	<0.001
Diastolic blood pressure (mmHg) (SD)	77.1 (26.2)	67.0 (25.3)	<0.001	79.7 (26.1)	67.1 (25.2)	<0.001
pH (SD)	7.139 (0.165)	7.047 (0.161)	<0.001	7.161 (0.151)	7.050 (0.165)	<0.001
Lactic acid (mmol/L) (SD)	9.435 (4.325)	11.265 (4.049)	<0.001	9.316 (4.466)	11.062 (4.043)	<0.001
White blood cell (count/µL) (SD)	13,250 (5469)	14,286 (21,284)	0.420	13,032 (5447)	14,246 (19,832)	0.290

Comparisons were made across the survivors (n = 224), non-survivors (n = 299), patients with favorable neurological outcomes (n = 173), and those with poor outcomes (n = 350). Key parameters include age, gender, health conditions, and clinical measures. Continuous variables are presented with the standard deviation (SD), and categorical data are indicated as percentages. A *p*-value < 0.05 indicates significant differences between groups.

### 3.3 CFS Distribution

**Fig. 2.**
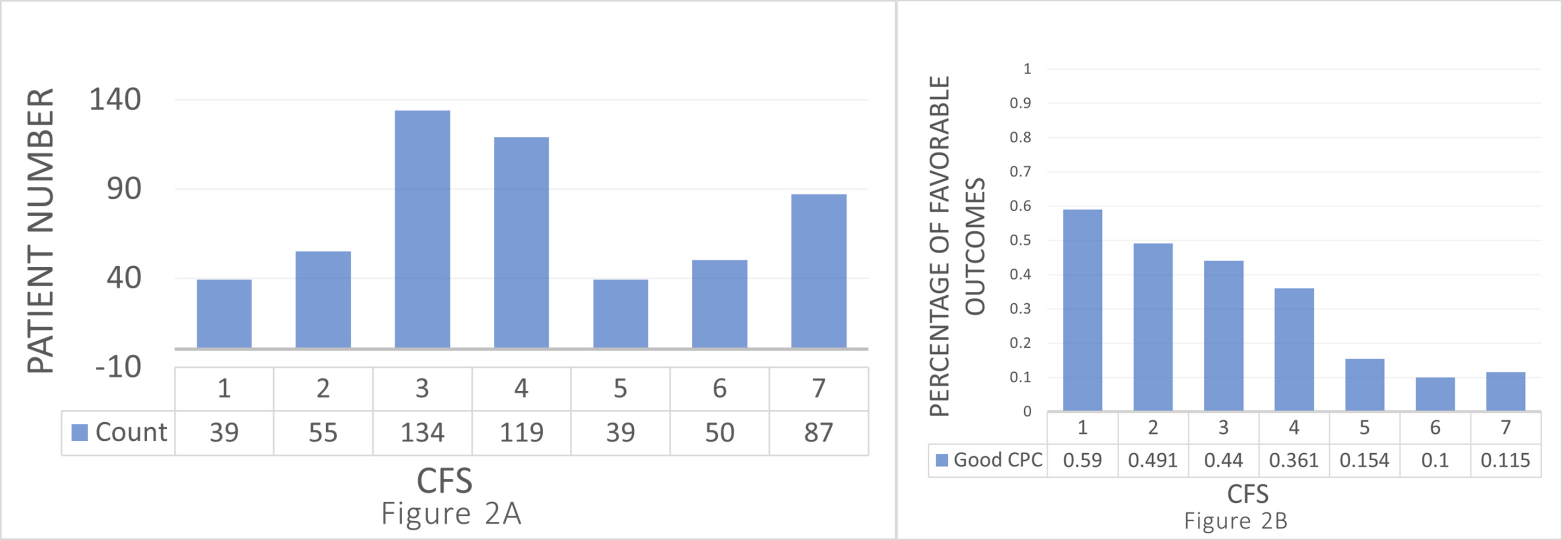
**Distribution and outcomes by Clinical Frailty Scale (CFS) scores**. (A) Number of patients for each CFS score. The distribution of individuals across different CFS categories ranges from 1 to 7. The distribution is as follows: CFS 1 (very fit), n = 39; CFS 2 (well), n = 55; CFS 3 (managing well), n = 134; CFS 4 (vulnerable), n = 119; CFS 5 (mildly frail), n = 39; CFS 6 (moderately frail), n = 50; CFS 7 (severely frail), n = 87. (B) Percentage of patients with good cerebral performance categories (CPC) for each CFS score. The percentage for good CPC (≤2) across different categories of the CFS ranges from 1 to 7. The percentage are as follows: CFS 1 (very fit): 59% (23/39), CFS 2 (well): 49% (27/55), CFS 3 (managing well): 44% (59/134), CFS 4 (vulnerable): 36% (43/119), CFS 5 (mildly frail): 15% (6/39), CFS 6 (moderately frail): 10% (5/50), CFS 7 (severely frail): 12% (10/87).

A total of 134 patients were classified with a CFS score of 3, while 119, 87, 55, 50, 39, and 39 were categorized with a CFS score of 4, 7, 2, 6, 1, and 5, respectively (Fig. 2A). The percentages of patients with favorable neurological outcomes by CFS scores are shown in Fig. 2B.

Patients with CFS 1 had the highest proportion of favorable neurological outcomes (59%, 23/39), followed by CFS 2 (49.1%, 27/55). In contrast, CFS 6 (10%, 5/50) and CFS 7 (11.5%, 10/87) presented the lowest percentages. The remaining 44% (59/134), 36.1% (43/119), and 15.4% (6/39) of patients with favorable neurological outcomes had a CFS score of 3, 4, and 5, respectively. Overall, the proportion of favorable outcomes decreased as the CFS scores increased.

**Fig. 3.**
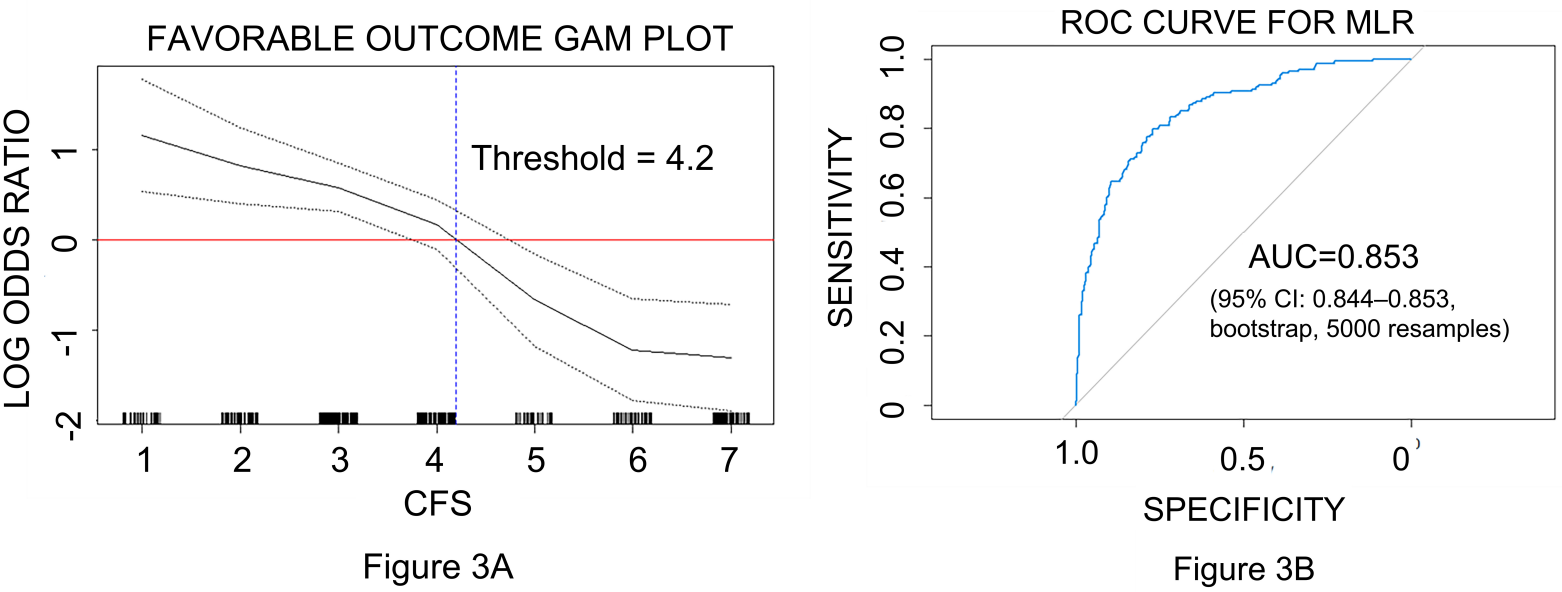
**Threshold analysis and model performance for predicting neurological outcomes**. (A) Generalized additive model for determining the threshold of the CFS. The generalized additive model (GAM) plot illustrates the association between CFS and favorable neurological outcomes. The inflection point at CFS = 4.2 corresponds to a log OR of 0. (B) Receiver operating characteristic curve (ROC) of the MLR model for association with good neurological outcomes. The ROC curve derived from the MLR model includes age, gender, CFS, witnessed collapse, pre-existing malignancy, underlying COPD or asthma, epinephrine dose during CPR, arterial blood gas pH, and DBP. The AUC of the model was 0.853, with a 95% confidence interval (CI) of 0.844–0.853, calculated using 5000 bootstraps resamples. CFS, Clinical Frailty Scale; COPD, chronic obstructive pulmonary disease; DBP, diastolic blood pressure; AUC, area under the curve; MLR, multiple logistic regression; CPR, cardiopulmonary resuscitation; OR, odds ratio.

The GAM plot in Fig. 3A shows the relationship between favorable neurological outcomes and the CFS.

At a CFS threshold of 4.2, a log odds ratio (OR) of 0 indicated no association between the CFS score and the likelihood of a favorable neurological outcome. Given the ordinal nature of the CFS, we identified that patients with favorable neurological outcomes had CFS scores <4.

### 3.4 Association between Independent Factors and Favorable Neurological Outcomes

The MLR model included factors such as age >70 years, male gender, CFS >4, witnessed collapse, pre-existing malignancy, underlying COPD or asthma, epinephrine dose during CPR, first arterial blood gas pH upon ICU admission, and the first post-ROSC DBP. A positive association was observed for the male gender (borderline significant), witnessed collapse, arterial blood gas pH >7.1, and DBP >65 mmHg. In contrast, age >70 years, CFS >4, pre-existing malignancy, underlying COPD or asthma (borderline significant), and epinephrine dose >2 mg were negatively associated with the outcome (Table 2).

**Table 2.** **Multiple logistic regression analysis model for favorable neurological outcomes**.

Variables	Odds ratio	95% CI	*p*-value
Age >70 years	0.969	0.953–0.986	0.0003
Gender (male)	1.638	0.958–2.842	0.0746
Clinical Frailty Scale score >4	0.301	0.163–0.540	<0.0001
Witnessed collapse	1.903	1.034–3.624	0.0434
Malignancy	0.421	0.209–0.813	0.0122
Chronic obstructive pulmonary disease/asthma	0.431	0.164–1.038	0.0712
Epinephrine >2 mg	0.776	0.712–0.840	<0.0001
pH >7.1	28.396	6.487–129.350	<0.0001
Diastolic blood pressure >65 mmHg	1.015	1.006–1.024	0.0015

This analysis evaluated factors influencing favorable neurological outcomes. Odds ratio (OR) >1 indicates positive associations, OR <1 indicates negative associations, and OR = 1 indicates no association. A *p*-value <0.05 signifies statistical significance. CI, confidence interval.

The MLR analysis identified CFS scores of ≤4 as independent predictors of favorable neurological outcomes (OR: 0.301, 95% CI: 0.163–0.540). The AUC of the MLR model was 0.853, with a 95% CI of 0.844–0.853, indicating the performance of the model is stable (Fig. 3B).

Representative cutoff points were selected across different sensitivity levels and their corresponding specificity and Youden index values, as summarized in Table 3. The clinical balance between sensitivity and specificity is optimized at a cutoff of 302, with a sensitivity of 0.8, a specificity of 0.771, and a maximum Youden index of 0.569. These findings provide a reference for selecting appropriate cutoff points to optimize diagnostic performance.

**Table 3.** **Cutoff points with sensitivity, specificity, and Youden index**.

Cutoff	Sensitivity	Specificity	Youden index
40	1.000	0.114	0.114
220	0.902	0.589	0.490
302*	0.798	0.771	0.569
344	0.699	0.843	0.542
384	0.601	0.909	0.510
408	0.503	0.929	0.431
436	0.399	0.957	0.356
462	0.301	0.983	0.283
481	0.202	0.989	0.191
501	0.098	0.994	0.093
519	0.000	1.000	0.000

Cutoff points represent different sensitivity levels corresponding to their cutoff values and specificity. The cutoff point marked with * indicates the maximum Youden index.

### 3.5 Subgroup Analysis 

**Fig. 4.**
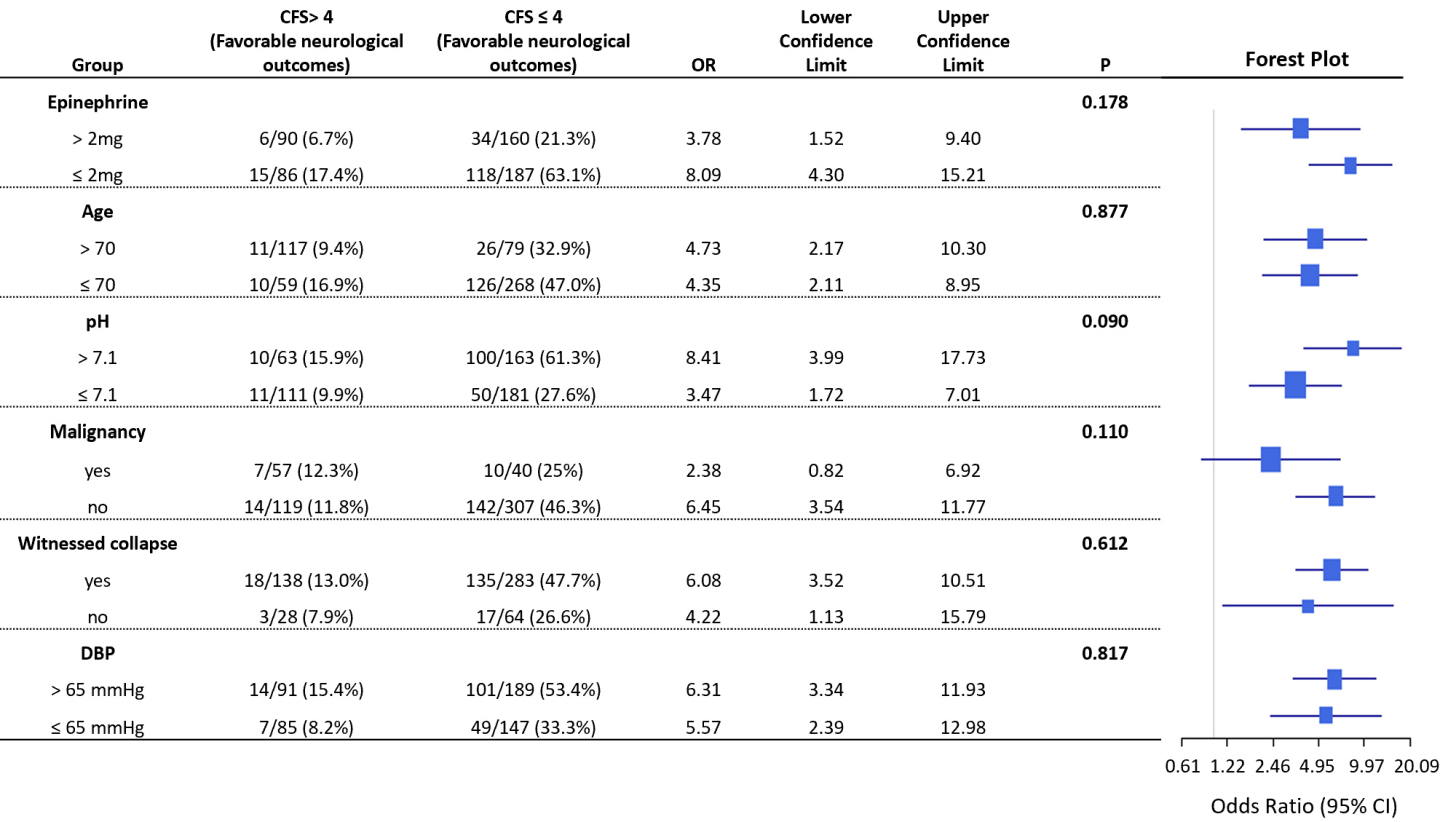
**Forest plot for analyzing the interaction between the CFS and other variables**. The forest plot illustrates potential interactions between the Cerebral Function Score (CFS) and significant variables in the multiple logistic regression (MLR) model. The plot investigates the interplay between CFS and factors such as epinephrine dose >2 mg (*p* = 0.178) during CPR, age (>70 years, *p* = 0.877), arterial blood gas pH (>7.1, *p* = 0.090), witnessed collapse (*p* = 0.612), malignancy (*p* = 0.110), and diastolic blood pressure (DBP >65 mmHg, *p* = 0.817); no significant interactions were observed. OR, odds ratio; CI, confidence interval; DBP, diastolic blood pressure; CPR, cardiopulmonary resuscitation.

A forest plot (Fig. 4) examined potential interactions between the CFS and significant variables identified in the MLR model. This analysis assessed whether the relationship between lower CFS scores and improved neurological outcomes was influenced by factors such as epinephrine dosage during CPR (>2 mg vs. ≤2 mg), age (>70 vs. ≤70), arterial blood gas pH (>7.1 vs. ≤ 7.1) upon ICU admission, malignancy, witnessed collapse, and the first DBP post-ROSC (>65 mmHg or ≤65 mmHg).

The analysis found no statistically significant interactions between the CFS and these variables. Specifically, no interaction was observed for an epinephrine dose of >2 mg (*p* = 0.178), age of >70 years (*p* = 0.877), an arterial blood gas pH of >7.1 (*p* = 0.090), malignancy (*p* = 0.110), witnessed collapse (*p* = 0.612), and DBP >65 mmHg (*p* = 0.817). These results suggest that the association between the CFS and neurological outcomes operates independently of these factors.

### 3.6 Correlation between CFS and Age

**Fig. 5.**
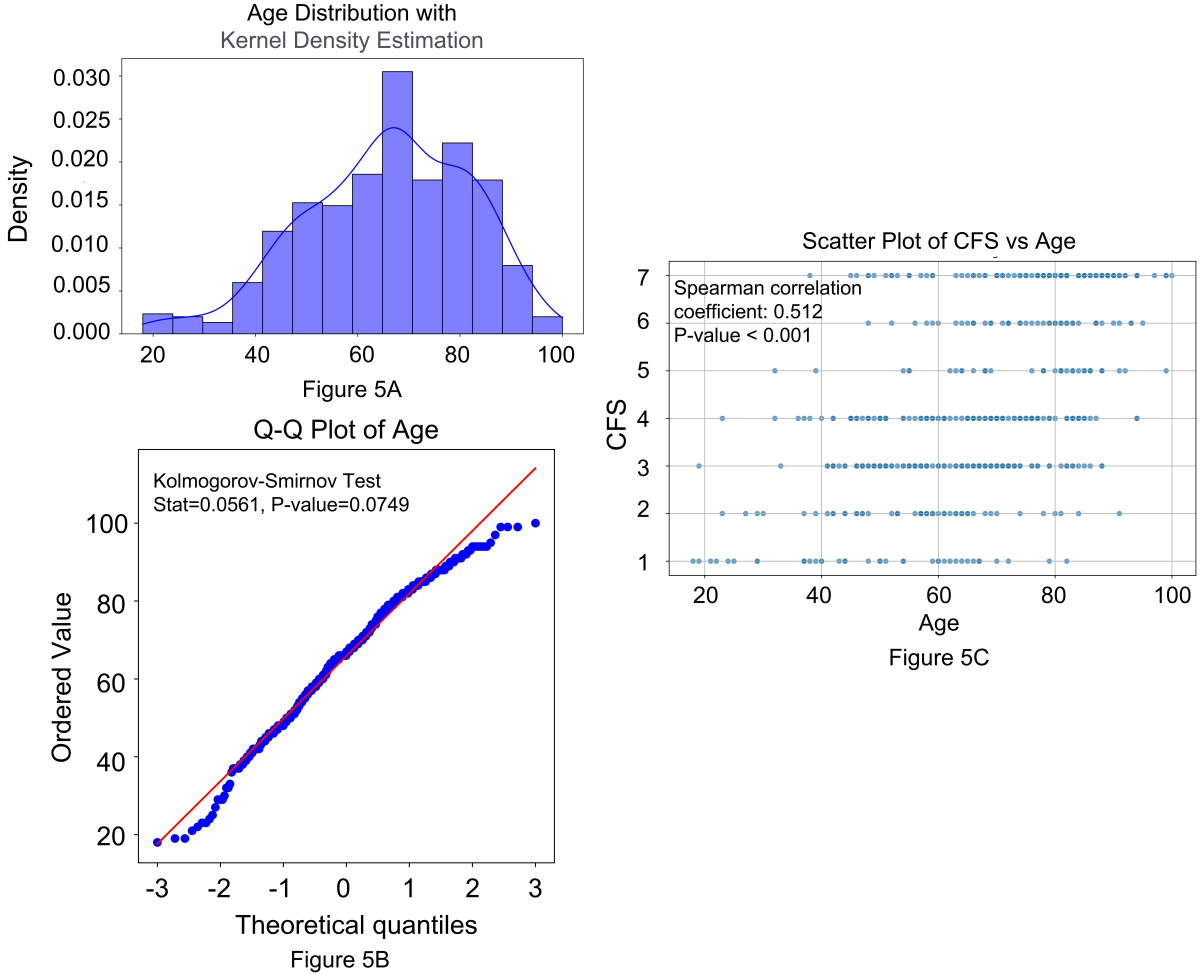
**Analysis of age distribution and its relationship with frailty**. (A) Age distribution with kernel density estimation (KDE). The histogram illustrates the distribution of age values in the dataset, while the KDE curve represents the smoothed probability density function. The x-axis shows age, and the y-axis shows density. (B) Q–Q plot of age. This Q–Q plot compares the quantiles of observed age data to a theoretical normal distribution. The red diagonal line represents the expected normal distribution, while the blue dots are the observed quantiles. The Kolmogorov–Smirnov test result (Stat = 0.0561, *p* = 0.0749) suggests that the age distribution follows approximate normality (*p* > 0.05). (C) Scatter plot of CFS vs. age. The scatter plot depicts the relationship between CFS and age. Each point represents an individual observation, with age on the x-axis and CFS on the y-axis. The Spearman’s correlation coefficient is 0.512 (*p* < 0.001), indicating a moderate positive correlation, where higher age is associated with increased frailty. CFS, Clinical Frailty Scale.

Fig. 5 illustrates the distribution of age and its relationship with CFS. The histogram (Fig. 5A) shows age distribution with a kernel density estimation (KDE) curve. The Q–Q plot (Fig. 5B) compares age quantiles to a theoretical normal distribution, with the Kolmogorov–Smirnov test suggesting approximate normality (*p* = 0.0749). A scatter plot (Fig. 5C) reveals a moderate positive correlation between age and CFS (Spearman’s coefficient = 0.512, *p* < 0.001), indicating that higher age is associated with increased frailty.

## 4. Discussion

This study demonstrated that higher CFS scores are significantly associated with worse neurological outcomes in OHCA patients and identified CFS scores >4 as independently associated with poor neurological recovery. However, no significant interactions were observed between CFS and age, epinephrine use, or a history of malignancy in predicting cardiac arrest outcomes.

### 4.1 Comparison between Pre-Existing Studies

The CFS has garnered increasing attention among resuscitation researchers for its effectiveness in predicting patient outcomes. A 2023 study by Yamamoto *et al*. [18] investigated the impact of frailty on neurological outcomes in ROSC patients using a nine-point CFS. While this study aligns with our focus, it differs in two key aspects: it included a larger cohort and used a nine-point scale instead of the seven-point scale employed in our study. Yamamoto *et al*. [18] predefined CFS scores ≥5 indicate high frailty. In contrast, our study used a GAM to establish an optimal CFS cutoff of 4. This difference may stem from variations in the scoring systems, with our cutoff of 4 aligning proportionally with their predefined cutoff of 5. Despite this, both studies confirm that higher CFS scores correlate with poorer neurological outcomes.

A study by Mowbray *et al*. [19], focusing on cardiac arrest survivors discharged to home care, found that severe frailty (CFS ≥5 of 9) was associated with an 8% decrease in 30-day survival odds per one-unit increase in CFS. However, they did not observe a significant relationship between higher CFS scores and functional or cognitive decline. These findings align with our hypothesis that higher CFS scores predict poorer neurological outcomes, though detailed assessments of very high CFS scores (8–9) may not always be necessary.

Similarly, McPherson *et al*. [13] observed that lower CFS scores were associated with improved survival and a higher prevalence of shockable rhythms in cardiac arrest patients. Shockable rhythm, indicative of cardiac-origin arrests, is also associated with better outcomes [20]. While our study excluded variables such as shockable rhythm and post-resuscitation care interventions (e.g., extracorporeal membrane oxygenation), further research is needed to explore whether optimal CFS cutoffs vary by cardiac arrest etiology and population demographics.

### 4.2 Distribution of CFS Scores in ROSC Patients

In our cohort, most ROSC patients had mid-range CFS scores, with a CFS score of 3 being the most frequent, followed by a CFS score of 4. This distribution reflects a specific subset of OHCA patients who survived resuscitation efforts. However, it may not represent the broader OHCA population, particularly given that CFS has not been validated in younger populations (<65 years), which form a substantial proportion of ROSC cases [19]. Additionally, higher CFS scores may correspond to greater cognitive impairments, such as dementia, which could independently affect neurological outcomes. Despite these limitations, our study highlights an inverse relationship between frailty and neurological outcomes, demonstrating that patients with higher CFS scores are less likely to achieve favorable outcomes.

### 4.3 Implications of a CFS Cutoff Point of 4

The GAM plot suggested a CFS score of 4 as the threshold for poor neurological outcomes. This finding supports the hypothesis that lower frailty levels are associated with better post-cardiac arrest outcomes. However, interpreting the CFS as an ordinal scale within a GAM introduces challenges, as the scale does not assume linear intervals. For example, the difference between a CFS score of 1 and 2 may not equate to that between a CFS score of 2 and 3; thus, a CFS score threshold of 4 should be viewed as a transitional zone rather than an exact cutoff.

### 4.4 Independent Factors Associated with Favorable Neurological Outcomes

Epinephrine dosage during CPR, reflecting resuscitation duration, significantly correlated with neurological outcomes. Our analysis selected epinephrine dosage as a representative variable. Both SBP and DBP also exhibited strong correlations with favorable outcomes. Consistent with prior findings, DBP outperformed SBP in predicting favorable neurological outcomes, with a lower cutoff value (>65 mmHg) in this study [21]. Additionally, COPD and asthma emerged as notable comorbidities influencing neurological outcomes, warranting further investigation despite their lack of statistical significance.

### 4.5 Integration of CFS with Other Independent Factors

Previous prediction models, such as the OHCA score (AUC 0.82–0.88), CAHP score (AUC 0.91–0.93), and CRASS score (AUC 0.88), have demonstrated robust performances in large cohorts [10, 11, 12]. While our study comprised a smaller sample size (n = 523), our model incorporating the CFS achieved a competitive AUC of 0.853. Notably, CFS scores >4 were negatively associated with favorable outcomes, with an OR of 0.301 (95% CI: 0.163–0.540). Although based on clinical judgment, the CFS is widely validated as a reliable tool and does not require complex measurements or laboratory tests [22]. Unlike traditional models, which often focus on resuscitation-related variables, including CFS, specific underlying diseases (e.g., malignancy, COPD/asthma) provide critical insight into the patient. Future predictive models could benefit from integrating the CFS to enhance performance and clinical applicability.

### 4.6 Proposed Biological Mechanisms

Frailty arises from multifactorial mechanisms, including impaired metabolism, cellular senescence, inflammation, hormone dysregulation, and immune dysfunction [23]. These processes overlap with those underlying brain recovery, which is equally multifaceted. Following OHCA, hypoxic-ischemic encephalopathy is the leading cause of poor neurological outcomes, characterized by hypoxia-induced cerebral dysfunction, inflammatory responses, apoptosis, cytotoxic edema, excitotoxicity, and seizures. These processes culminate in cell death and brain remodeling, reducing neuronal excitability [24]. Frail patients with pre-existing physiological dysregulation may face additional challenges in recovery; thus, further research into the shared mechanisms of frailty and brain recovery is essential.

### 4.7 Implications of the Association between Age and CFS

Frailty predictably correlates with age, as evidenced by a moderate positive correlation (Spearman’s coefficient = 0.512). However, our MLR analysis revealed that age and CFS independently influence neurological outcomes, underscoring their distinct prognostic roles. Biologically, frailty arises from an interplay between aging and disease mechanisms, contributing to disability, dependency, and mortality [23]. Therefore, incorporating age and frailty into clinical assessments provides a more comprehensive evaluation of patient prognosis.

### 4.8 Limitations

This study has several limitations. First, the single-center, retrospective design, conducted at a metropolitan tertiary center with advanced interventions such as targeted temperature management and extracorporeal membrane oxygenation, may limit generalizability. Our model was also not externally validated. Second, the retrospective determination of CFS through medical record review introduces potential bias. Third, the relatively small sample size affects statistical power. Fourth, while the GAM identified a CFS threshold, its linearity assumptions are challenged by the ordinal nature of the CFS. Fifth, excluding patients with DNR orders, who are typically frailer, may underestimate the broader impact of frailty. Finally, our study did not explore the relationship between cardiac arrest etiology and premorbid CFS, which could influence results.

### 4.9 Future Directions

Future studies should adopt multicenter, prospective designs with larger, diverse populations to enhance generalizability. Investigating the biological mechanisms linking frailty and neurological outcomes and incorporating biomarkers or imaging techniques could improve prognostic accuracy. Studies examining frail patients with DNR orders would further elucidate the impact of frailty in broader care contexts.

## 5. Conclusions

This study identified higher CFS scores as independently associated with worse neurological outcomes in OHCA patients with ROSC. Prospective multicenter studies are needed to validate the prognostic utility of the CFS.

## Availability of Data and Materials

The datasets generated and/or analyzed during the current study are not publicly available due to patient privacy protection. However, they are available from the corresponding author on reasonable request.
